# High Temporal Resolution Parametric MRI Monitoring of the Initial Ischemia/Reperfusion Phase in Experimental Acute Kidney Injury

**DOI:** 10.1371/journal.pone.0057411

**Published:** 2013-02-28

**Authors:** Andreas Pohlmann, Jan Hentschel, Mandy Fechner, Uwe Hoff, Gordana Bubalo, Karen Arakelyan, Kathleen Cantow, Erdmann Seeliger, Bert Flemming, Helmar Waiczies, Sonia Waiczies, Wolf-Hagen Schunck, Duska Dragun, Thoralf Niendorf

**Affiliations:** 1 Berlin Ultrahigh Field Facility, Max Delbrück Center for Molecular Medicine, Berlin, Germany; 2 Nephrology and Intensive Care Medicine, Charité Campus Virchow-Klinikum and Center for Cardiovascular Research, Charité, Berlin, Germany; 3 Institute of Physiology, Center for Cardiovascular Research, Charité-Universitätsmedizin Berlin, Berlin, Germany; 4 Experimental and Clinical Research Center, a joint cooperation between the Charité Medical Faculty and the Max Delbrück Center for Molecular Medicine, Berlin, Germany; 5 Max Delbrück Center for Molecular Medicine, Berlin, Germany; Osaka University Graduate School of Medicine, Japan

## Abstract

Ischemia/reperfusion (I/R) injury, a consequence of kidney hypoperfusion or temporary interruption of blood flow is a common cause of acute kidney injury (AKI). There is an unmet need to better understand the mechanisms operative during the initial phase of ischemic AKI. Non-invasive *in vivo* parametric magnetic resonance imaging (MRI) may elucidate spatio-temporal pathophysiological changes in the kidney by monitoring the MR relaxation parameters T_2_* and T_2_, which are known to be sensitive to blood oxygenation. The aim of our study was to establish the technical feasibility of fast continuous T_2_*/T_2_ mapping throughout renal I/R. MRI was combined with a remotely controlled I/R model and a segmentation model based semi-automated quantitative analysis. This technique enabled the detailed assessment of *in vivo* changes in all kidney regions during ischemia and early reperfusion. Significant changes in T_2_* and T_2_ were observed shortly after induction of renal ischemia and during the initial reperfusion phase. Our study demonstrated for the first time that continuous and high temporal resolution parametric MRI is feasible for *in-vivo* monitoring and characterization of I/R induced AKI in rats. This technique may help in the identification of the timeline of key events responsible for development of renal damage in hypoperfusion-induced AKI.

## Introduction

Acute kidney injury (AKI) can be caused by kidney hypoperfusion or temporary interruption of blood flow in various clinical settings [1]–[3]. This ischemia/reperfusion injury (I/RI) is characterized by mismatch of local tissue oxygen supply and local cellular energy demand and results in sublethal or lethal injuries to the tubular epithelium and neighbouring cell structures which is clinically detected as compromised glomerular filtration rate [4], [5]. Despite substantial progress in the field of AKI including biomarker discovery and renal replacement therapy dosing, there is an unmet need to better understand the mechanisms operative during the initial phase of ischemic AKI. Treatments targeting processes operative before the onset of sublethal or lethal cellular injuries would have great clinical impact. Animal models are well suited to study mechanisms involved in the pathogenesis of renal ischemia/reperfusion injury and helped to define the injury phases of initiation, extension and regeneration [6]–[8]. The imbalance between oxygen demand and supply is considered to be the initiating step in the pathophysiologic cascade of events [9]–[11]. Renal tissue oxygen tension (pO_2_) as determined by invasive *in-vivo* measurements in the first hour after reperfusion may be decisive for renal damage at 48 hours [12], [13]. However, lack of adequate diagnostic tools capable to determine spatio-temporal distribution of oxygenation during the phases of renal ischemia/reperfusion precluded elucidation of events taking place immediately after initiation of ischemia and reperfusion.

Non-invasive *in vivo* imaging holds the promise to elucidate spatio-temporal pathophysiological changes in the kidney by monitoring the early phases of ischemia and reperfusion. Magnetic resonance imaging (MRI) meets the needs of non-invasive renal *in-vivo* imaging. Parametric MRI can provide maps of the MR relaxation parameters T_2_* and T_2_, which are known to be blood oxygenation level-dependent (BOLD) [13]. The BOLD effect relies on the paramagnetic property of deoxygenated hemoglobin. A signal attenuation in T_2_*-weighted MR images occurs if the volume fraction of deoxygenated hemoglobin increases. T_2_* or its reciprocal value (R_2_* = 1/T_2_*) have been used in numerous clinical and preclinical studies as a surrogate of renal (blood) oxygenation [14]. Renal R_2_* has even been suggested to display a close correlation with renal tissue pO_2_ levels [15], and R_2_*-mapping has been deemed ideally suited for the evaluation of intra-renal oxygenation [14]. The full potential of BOLD MRI and T_2_*/T_2_ mapping remained as yet untapped [16]–[20]. Few rodent studies performed so far include preliminary reports about T_2_*/T_2_ measurements in renal ischemia models [21]–[23], chronic renal artery stenosis in rats [16] and ischemia/reperfusion (I/R) in mice [20]. Due to technical limitations, none of these studies focused on high temporal resolution continuous parametric MRI based *in-vivo* monitoring during ischemia – and in particular during early reperfusion of the kidney. We sought to establish the feasibility high temporal resolution longitudinal T_2_*/T_2_ mapping throughout I/R. To meet this goal an I/R model that affords remote induction of ischemia/reperfusion inside an MR scanner is implemented together with a standardized segmentation model of the rat kidney. This integrated approach enabled for the first time the monitoring of renal T_2_* and T_2_ in the initial reperfusion phase.

## Materials and Methods

### Ethics Statement

Animal experiments were carried out in accordance with the guidelines provided and approved by the Animal Welfare Department of the *Landesamt für Gesundheit und Soziales* Berlin (Berlin State Office of Health and Social Affairs, permit numbers G00121/11, G0059/12). All experiments were carried out under urethane anesthesia (20% in saline, 6 ml/kg body mass, intra-peritoneal), and all efforts were made to minimize suffering. Animals were kept under constant thermal conditions: 37°C body temperature was maintained by warming the animal beds using a circulating heated water system (Thermo Haake GmbH, Karlsruhe, Germany). Rectal temperature and respiratory motion/rate were monitored (SA Instruments, Inc., New York, USA) throughout the imaging experiments.

### MR Imaging

All imaging experiments were carried out on a 9.4T small animal MR system (Biospec 94/20, Bruker Biospin, Ettlingen, Germany) equipped with a linear polarized birdcage RF resonator for transmission in conjunction with a curved four channel receive RF coil array (Bruker Biospin, Ettlingen, Germany) customized for rats.

MRI protocols for parametric mapping of T_2_* and T_2_ were tailored for fast measurements in the rat kidney at a magnetic field strength of 9.4 Tesla. T_2_-weighted pilot scans and local B_0_ shimming that uses a voxel enclosing the kidney only were performed first. Subsequently, parametric T_2_* and T_2_ mapping using respiratory gated imaging techniques was performed. For T_2_* mapping a multi gradient echo (MGE) sequence (repetition time = 50 ms, echo times = 10, first echo time = 1.43 ms, echo spacing 2.14 ms, averages = 4) was employed with a total acquisition time of approx. 1 min 20 s (respiratory rate dependent). For T_2_ mapping a multi spin echo (MSME) sequence (repetition time = 550 ms, echo times = 7, first echo time = 10 ms, echo spacing 10 ms, averages = 1) was used with a total acquisition time of approx. 1 min 40 s. A coronal oblique image slice was acquired with a spatial in plane resolution of (226×445) µm^2^ (field of view = (38.2×50.3) mm^2^, matrix size = 169×113 zero-filled to 169×215) and a slice thickness of 1.4–1.5 mm.

Renal blood flow during I/R experiments was monitored by time-of-flight (TOF) MR angiographies of the kidney. TOF angiography consisted of an untriggered spoiled gradient echo sequence (2D FLASH, repetition time = 11 ms, echo time = 3 ms, flip angle = 80 degree, spatial in plane resolution of (200×268) µm^2^) with 15 slices of 1.0 mm thickness placed perpendicular to the major renal blood vessels, acquired in 24 seconds.

### Image Analysis and Kidney Segmentation Model

Parametric maps of absolute T_2_* and T_2_ values were calculated by pixel-wise mono-exponential fitting to the signal intensities of the T_2_*- and T_2_-weighted images acquired at different echo times (in-house developed program; MATLAB, R2010a, MathWorks, Natick, WA, USA). The relaxation times T_2_* and T_2_ were chosen over the relaxivities (i.e. R_2_* = 1/T_2_* and R_2_ = 1/T_2_), as they allow for a better visualization of changes in the parameter maps for the different kidney regions and stimuli. In quantitative data representations such as value tables or bar charts the relaxation times are complemented by the corresponding relativities, which were also derived on a pixel-by-pixel basis.

Movements of the kidney during I/R experiments were compensated by image registration (FLIRT, FSL, www.fmrib.ox.ac.uk/fsl) using the first echo images of the multi-echo MGE or MSME acquisitions. Images were registered onto a baseline scan and the resulting spatial transformation matrices applied to the corresponding parameter maps.

**Figure 1:**
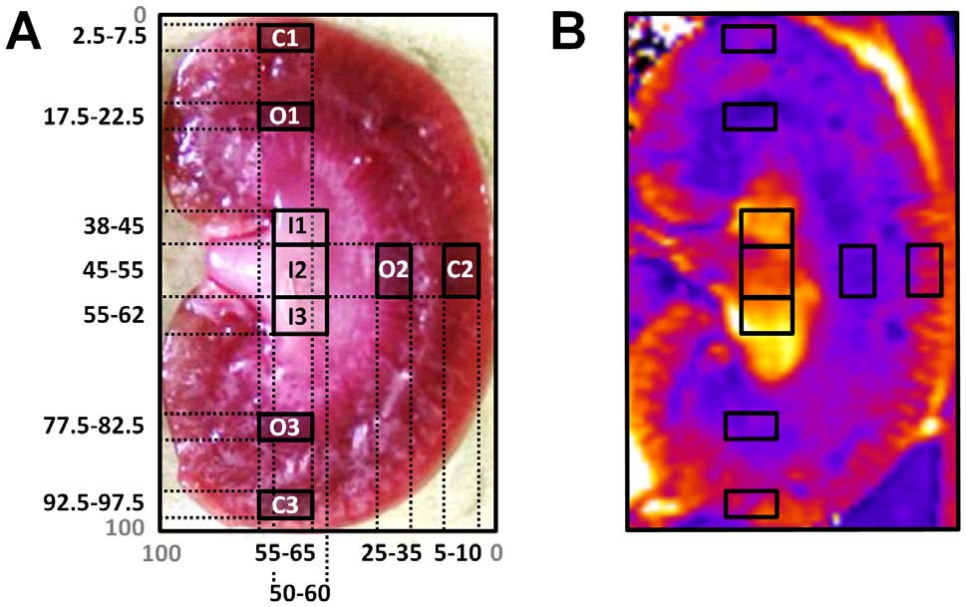
Standardized segmentation model of the rat kidney that is used to place regions-of-interest (ROI) in the cortex (C1,C2,C3), the outer medulla (O1,O2,O3) and the inner medulla (I1,I2,I3). **A)** Segmentation model overlaid onto a photograph of a freshly excised coronal view of a rat kidney. During analysis the rectangular reference frame is manually positioned around the kidney in the coronal view. The ROIs are placed automatically at pre-defined relative positions within this reference frame (number ranges signify percentages of the reference frame dimensions). **B)** Example of a color-coded T_2_* parameter map, showing a rat kidney in-vivo together with an overlay of the segmentation model.

Mean T_2_* (R_2_*) and T_2_ (R_2_) values for several regions-of-interest (ROI) within the renal cortex, outer medulla and inner medulla were calculated from the parameter maps. To reduce operator-induced variability in the ROI placement a standardized segmentation model of the rat kidney was developed. For this purpose special care was taken with regard to the location of the ROIs in the kidney. The aim was to determine ROIs strictly according to morphological kidney features, i.e., the distinct renal layers: cortex, outer medulla, and inner medulla (*Figure 1*). To this end, the dimensions of the rat kidney layers were measured in a series of freshly harvested kidneys as well as in a series of formalin-fixed kidneys (altogether 16 kidneys). To account for the inter-individual variability of the absolute length (cranial to caudal extremities) and width (lateral to medial border) of the kidneys, a rectangular frame that tightly encloses the kidney in the coronal view was used as a reference. The segmentation model consists of nine ROIs at defined relative positions (percentages of both diameters) within this rectangular reference frame (*Figure 1*). The size and positions of nine ROIs were defined for three ROIs in the renal cortex (COR), for three ROIs in the outer medulla (OM), and for three ROIs in the inner medulla (IM). All ROIs were placed in safe distance from the borders between these kidney layers to avoid any ‘contamination’ from the neighboring layers (partial segment effects) and to allow for inter-individual variations in morphology without the need to change the ROI position. The implementation of this model in a semi-automated analysis program developed in ImageJ (NIH, Bethesda, MD, USA, http://imagej.nih.gov/ij) limited user interaction to the placement of the rectangular reference frame around the kidney. The automatically placed ROIs were overlaid onto the parameter maps and their position was visually validated. ROIs that included artifacts were excluded from further analysis.

Due to the need for respiratory triggering the time intervals between MRI scans (∼180 seconds) varied slightly during the imaging experiments and between animals. To allow for a comparison between different animals the acquired T_2_* time courses were linearly interpolated and T_2_* calculated for time points at exact intervals of 180 seconds. Group mean values and standard errors of the mean (SEM) were then calculated for each time point and T_2_* (mean ±SEM averaged over six animals) plotted versus time. In all other cases (e.g. bar charts of parameter values at end-baseline, end-ischemia, etc) the original, non-interpolated, data were used.

### Hypoxia and Hyperoxia Experiments

Renal T_2_* and T_2_ mapping at 9.4 Tesla in rats under variable physiological conditions has not been reported so far. Hence, the sensitivity of T_2_* and T_2_ to renal blood oxygenation changes was demonstrated by examining the changes induced in these parameters by externally controlled variations of blood oxygenation. Blood oxygenation level changes (hypoxia or hyperoxia) were induced in six male rats (Wistar, aged 3–4 month, 300–350 g body weight) by adapting the inhaled gas composition.

#### Experimental protocol

**Figure 2:**
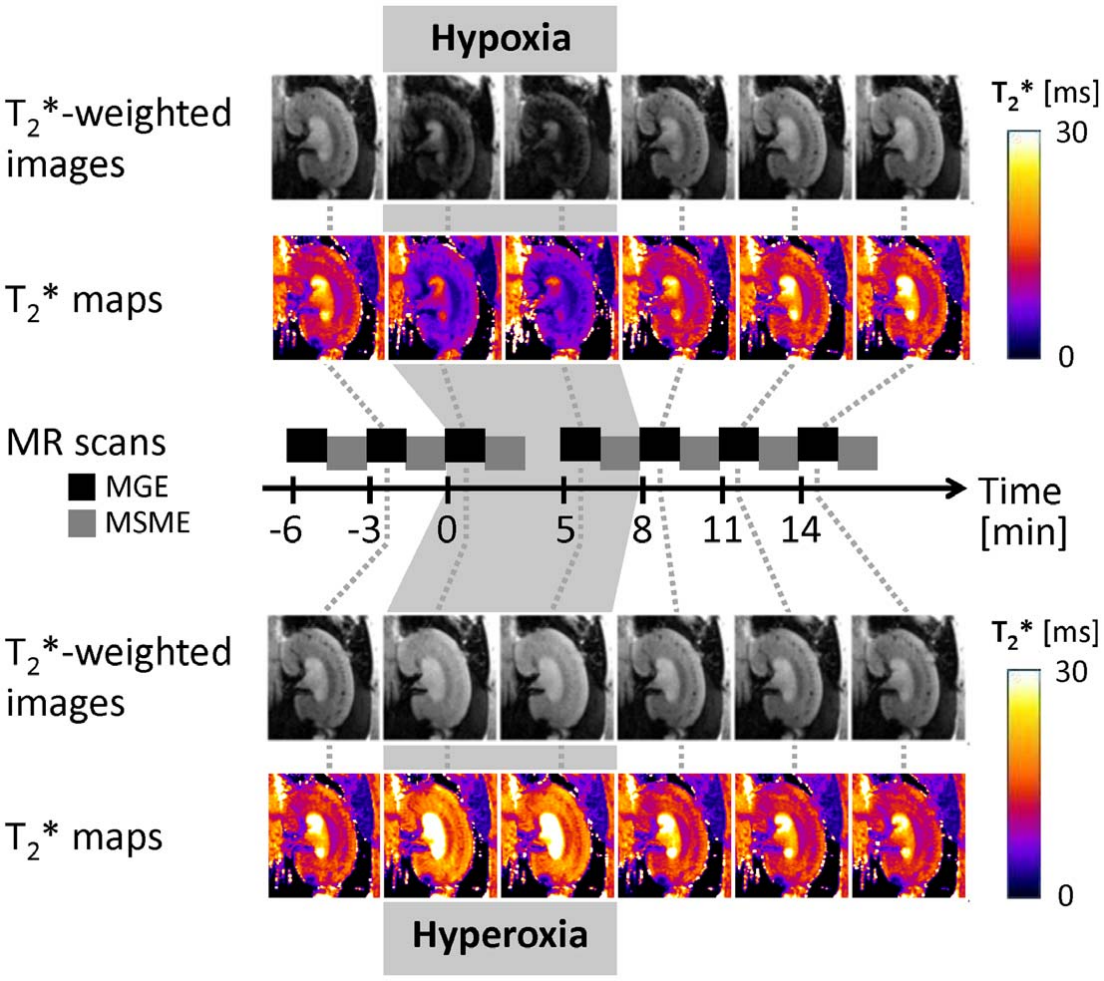
Hypoxia and hyperoxia experiments: T_2_*-weighted MR images (gray scale) and T_2_* parameter maps (color-coded) derived from MGE acquisitions. Following two baseline measurements of T_2_*, hypoxia/hyperoxia was induced by changing the inhaled gas mixture from air to 10%/90% O_2_/N_2_ or 100% O_2_. T_2_*/T_2_ measurements were repeated directly after and exactly 5 minutes after onset of hypoxia/hyperoxia (to ensure equal timing of the MR scans). Subsequently inhaled gas was changed back to air and T_2_*/T_2_ monitored continuously for another ∼9 minutes.

A respiratory mask was used, which provided air (normoxia) at a flow rate of 1000 ml/min. Following baseline measurements of T_2_*/T_2_, hypoxia was induced by adjusting the gas mixture to 10/90% O_2_/N_2_. T_2_*/T_2_ measurements were conducted throughout 8 minutes after induction of hypoxia. The final T_2_*/T_2_ scans during hypoxia were started exactly 5 minutes after hypoxia onset, to ensure equal hypoxia durations and comparable timing of the MR scans. On completion of these MR scans, inhaled gas mixture was changed back to air and T_2_*/T_2_ continuously monitored for another ∼9 minutes. The same protocol was followed for the hyperoxia experiments by setting the inhaled gas to 100% O_2_. *Figure 2* illustrates the experimental procedure, by showing the timeline and the corresponding T_2_*-weighted MR images together with calculated T_2_* parameter maps acquired prior, during and after hypoxia/hyperoxia.

### Ischemia/Reperfusion Experiments

Six male rats (Lewis, aged 2–3 months, 250–300 g body weight) underwent experimental ischemia-reperfusion inside the MR scanner.

#### Preparation

**Figure 3:**
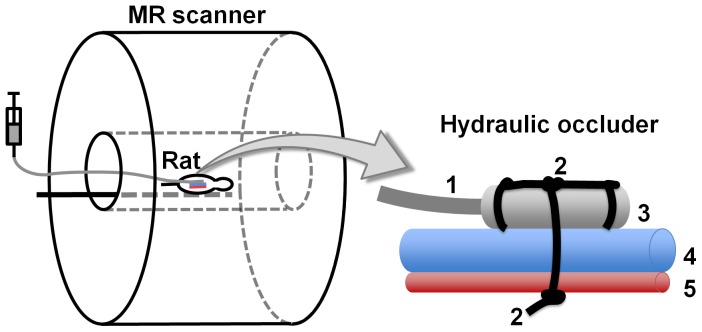
Schematic view of the hydraulic occluder (right) used for induction of renal ischemia during MRI. 1 = indistensible extension tube, 2 = sutures, 3 = distensible silicone tube, 4 = renal vein, 5 = renal artery. A water-filled syringe, connected to the indistensible extension tube, is used to create a hydraulic pressure, which leads to an inflation of the distensible tube. This causes a compression of the renal artery and vein and restricts the blood flow.

The abdominal cavity was opened by a midventral incision and the right kidney was removed. A remote controlled hydraulic occluder was placed around the renal artery and vein of the remaining left kidney to allow induction of renal ischemia. The hydraulic occluder (*Figure 3*) consists of a distensible silicone tube that is connected to a syringe via an indistensible extension tube. A suture loop around the distensible tube and renal vein and artery leads to a compression of the artery and vein when the distensible tube is inflated hydraulically. For intra-arterial administration of a vehicle solutions (to be replaced by drugs in future studies) to the kidney a catheter was placed in the aorta with its tip directly at the renal branch. A second remote controlled hydraulic occluder was placed just below the renal branch around the aorta. With the purpose of monitoring the temperature of the kidney throughout the imaging experiment, a fiber-optic temperature probe (OTP-M, AccuSens, Opsens, Quebec City, Canada) was positioned in close proximity to the kidney. The abdominal cavity was then filled with warmed saline and closed by sutures.

#### Experimental protocol

**Figure 4:**
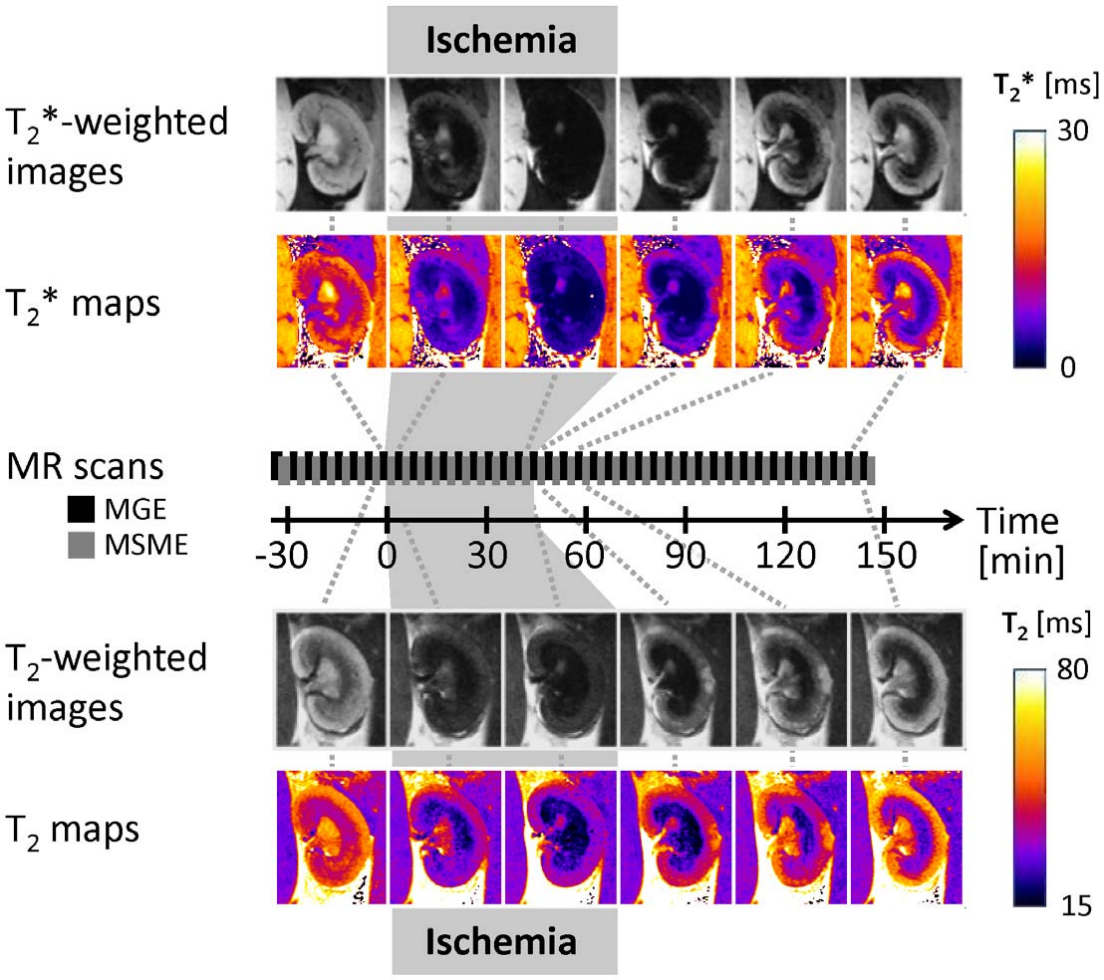
Ischemia reperfusion experiments: T_2_*-weighted (MGE acquisition) and T_2_-weighted MR images (MSME acquisition) shown in gray scale together with color-coded T_2_* and T_2_ parameter maps. Alternating T_2_* and T_2_ measurements (MGE and MSME scans) were repeated continuously throughout the entire experiment (55 time points). Images and maps are shown for six time points: last baseline, onset of ischemia and end of ischemia after 45 minutes, onset of reperfusion, and 12 minutes and 100 minutes after onset of reperfusion. All parameter maps are shown in Figure S2.

Directly following surgery the animal was transferred to the warmed MRI animal bed. For the remainder of the experiment T_2_* and T_2_ were continuously monitored by interleaved T_2_*/T_2_ mapping with a temporal resolution of ∼3 minutes. After a baseline of five T_2_*/T_2_ measurements the hydraulic occluder around the aorta was temporarily closed and a vehicle (100 µl of saline containing 1% DMSO) administered. Thereafter, ischemia was induced by closing the hydraulic occluder around the renal artery and vein for 45 minutes, followed by a reperfusion phase of ∼100 minutes. Renal blood flow during the three phases of the I/R experiment was monitored by time-of-flight (TOF) MR angiographies of the kidney, acquired prior and immediately after ischemia induction, as well as after onset of reperfusion. The absence of the flow-dependent intra-vascular signal during ischemia served to confirm complete occlusion of the renal artery and vein. *Figure 4* illustrates the experimental procedure, by showing the timeline and the corresponding T_2_*-weighted and T_2_-weighted MR images for six time points, together with T_2_* and T_2_ parameter maps.

### Statistical Analysis

T_2_* and T_2_ derived from the nine kidney ROIs were statistically analyzed using SPSS (version 20, IBM Deutschland GmbH, Germany). Data are expressed as mean±SEM. Normal distribution was confirmed by a one-sample Kolmogorov-Smirnov test. Tests of significance (2-tailed t-test) were performed for the last time points in each experimental phase (baseline, ischemia, reperfusion) following Levene’s test for equality of variances. A probability value of ≤0.05 was considered significant.

## Results

Changes in renal T_2_*, T_2_ were explored during variations of inspiratory gas composition (hypoxia, hyperoxia) and during renal ischemia/reperfusion.

### Hypoxia and Hyperoxia Experiments

The variations in inspiratory gas composition demonstrated a high sensitivity of T_2_* and T_2_ to renal blood oxygenation changes. Parametric mapping revealed a decrease in T_2_* and T_2_ for hypoxia and an increase for hyperoxia in all kidney layers (*Fig. 2*). For quantitative examination of T_2_* and T_2_ changes, mean values were determined for nine regions-of-interest (ROI) according to the proposed standardized renal segmentation model. Under hypoxic conditions T_2_* decreased by 35–49% (COR), 53–62% (OM), and 53–64% (IM), while T_2_ decreased by 25–29% (COR), 29–33% (OM), and 29–31% (IM). The observed increase during hyperoxia was 18–23% (COR), 31–42% (OM), 38–45% (IM) for T_2_* and 3–8% (COR), 11–20% (OM), 11–16% (IM) for T_2_.

### Ischemia/Reperfusion Experiments

T_2_* and T_2_ were monitored before and during ischemia/reperfusion with a temporal resolution of ∼3 minutes. *Figure 4* shows T_2_*-weighted and T_2_-weighted MR images for six time points, together with T_2_* and T_2_ parameter maps. The six time points represent last baseline, onset of ischemia and end of ischemia after 45 minutes, onset of reperfusion, and 12 minutes and 100 minutes after onset of reperfusion. T_2_* and T_2_ parameter maps of an entire ischemia reperfusion experiment are presented in Figures S1 and S2. These parametric maps demonstrated immediate changes in T_2_*/T_2_ after onset and end of ischemia.

**Figure 5:**
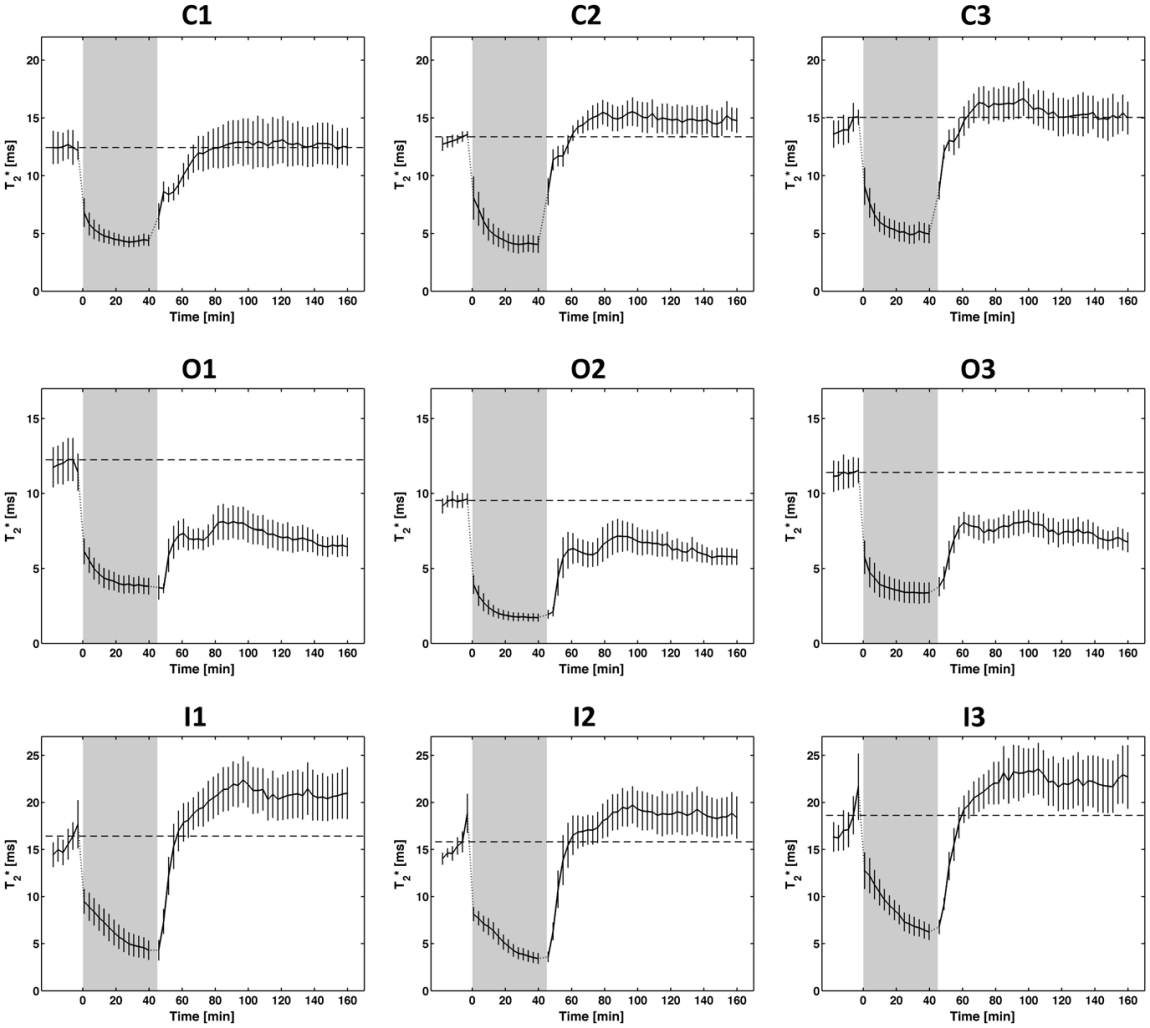
Ischemia reperfusion results derived from the standardized segmentation model of the kidney: Plots of T_2_* (mean ±SEM averaged over six animals) versus time for the ROIs in the cortex (C1, C2, C3; top row), in the outer medulla (O1, O2, O3; middle row), and in the inner medulla (I1, I2, I3; bottom row). Ischemia (shaded in gray) led to an immediate and significant T_2_* decrease in all kidney ROIs (p≤0.01 in C1, p≤0.001 in all other ROIs). Clear differences between cortex, outer medulla and inner medulla were observed. At the end of the reperfusion period T_2_* was close to baseline (dashed line) in the cortex (p>0.2), below baseline in the outer medulla (p≤0.05 (C1), p≤0.001 (C2), p≤0.01 (C3)), and above baseline in the inner medulla (non-significant). The three ROIs within each kidney regions showed very similar but not identical trends.

Quantitative examination of T_2_* and T_2_ changes used the standardized renal segmentation model. Plots of T_2_* (mean ±SEM averaged over six animals) versus time for these ROIs are shown in *Figure 5*. The T_2_* time courses revealed an immediate drop in T_2_* after onset of ischemia followed by a slow and steady decrease in T_2_*. During ischemia T_2_* was significantly reduced in all ROIs (p≤0.01 in C1, p≤0.001 in all other ROIs), which clearly distinguished this condition from baseline. The time courses of T_2_ were very similar to those of T_2_*. During reperfusion, T_2_* and T_2_ did return to baseline in the cortex, but not in the medulla. While no major differences among the cortical, outer medullary, and inner medullary T_2_* changes were found during ischemia, there was a clear regional dynamics during reperfusion. After onset of reperfusion T_2_* returned close to baseline for cortical segments (p = 0.97 (C1), p = 0.22 (C2), p = 0.65 (C3)). For the outer medulla T_2_* remained below baseline throughout reperfusion and was at 40–47% below baseline after 100 minutes (p≤0.05 (C1), p≤0.001 (C2), p≤0.01 (C3)). T_2_ was 19–21% below baseline at 100 minutes reperfusion. In the inner medulla T_2_* and T_2_ exceeded baseline by 17–28% and 14–18% respectively (non-significant) at the end of reperfusion. These differences among the layers were pronounced during early reperfusion and remained stable throughout reperfusion. The assessment of the heterogeneity among segments within one kidney layer revealed an almost equal time course in T_2_* for the cortical, outer medullary and inner medullary segments, respectively.

### Comparison of Hypoxia/Hyperoxia and Ischemia/Reperfusion

**Figure 6:**
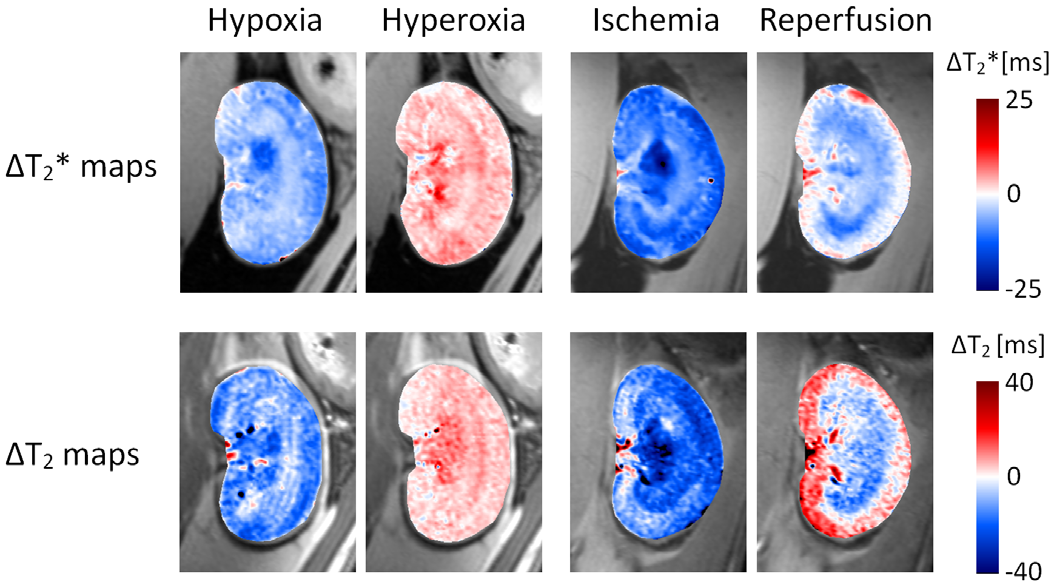
Change of renal T_2_* and T_2_ during hypoxia, hyperoxia, ischemia and reperfusion. Shown are T_2_* and T_2_ difference maps of the kidney (color-coded, overlay on anatomical MR image) between the last time point in each experiment phase and baseline. The three interventions (hypoxia, hyperoxia, ischemia) led to a reasonably homogeneous decrease/increase in both parameters. During ischemia the magnitude of the T_2_*/T_2_ reduction exceeded that during hypoxia. Parameter changes in the reperfusion phase clearly differentiated cortex and medulla: while medullary T_2_*/T_2_ remained reduced throughout reperfusion, in the cortex T_2_* returned to baseline and T_2_ rose above baseline.

Changes in renal T_2_* and T_2_ during hypoxia, hyperoxia, ischemia and reperfusion are illustrated in *Figure 6* by T_2_* and T_2_ difference maps. Hypoxia/hyperoxia led to a rather uniform T_2_*/T_2_ reduction/rise across the kidney. The T_2_*/T_2_ reduction in ischemic kidneys exceeded that during hypoxia. Changes in the course of reperfusion clearly differentiated cortex and medulla. In accordance with the ROI data medullary T_2_*/T_2_ remained reduced throughout reperfusion, cortical T_2_* returned to baseline and cortical T_2_ even rose markedly above baseline.

**Figure 7:**
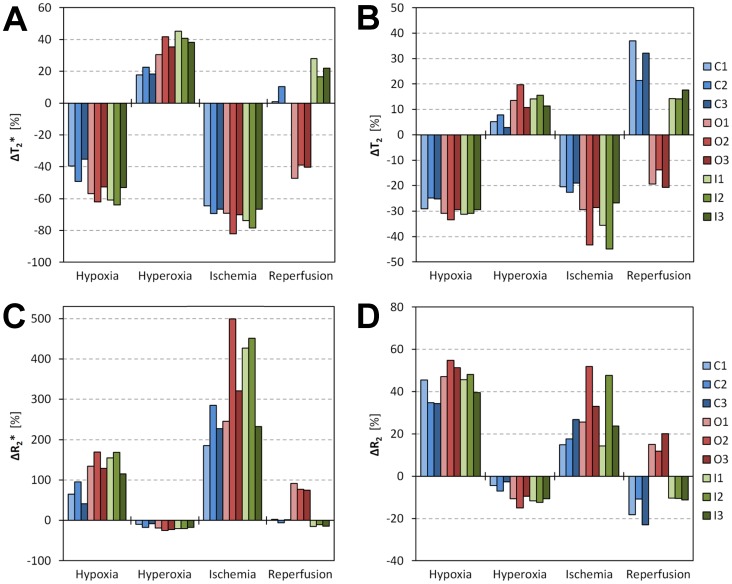
Synopsis of the hypoxia/hyperoxia and ischemia/reperfusion results. Mean changes ΔT_2_* (a), ΔT_2_ (b), ΔR_2_* (c) and ΔR_2_ (d) in percent of baseline for all regions-of-interest in the cortex (C1, C2, C3; blue), in the outer medulla (O1, O2, O3; red) and inner medulla (I1, I2, I2; green). Shown are the values for end-hypoxia, end-hyperoxia, end-ischemia and end-reperfusion.

For a quantitative synopsis of the ischemia-reperfusion results together with the hypoxia/hyperoxia results the MR parameter values at baseline, end-hypoxia/hyperoxia, end-ischemia and end-reperfusion were chosen. The group mean T_2_* and T_2_ with standard errors (SEM) are surveyed for all regions-of-interest in Table S1, together with the corresponding relativities R_2_* and R_2_. Relative changes in these parameters are summarized in *Figure 7*. ΔT_2_* (ΔR_2_*) and ΔT_2_ (ΔR_2_) derived from the outer/inner medulla exceeded those observed in the cortex (except for ΔT_2_ (ΔR_2_) during reperfusion). For all regions the T_2_* (R_2_*) changes during ischemia were more pronounced than during hypoxia. T_2_ (R_2_) effects during ischemia were comparable to hypoxia.

## Discussion

Our study demonstrates that continuous and high temporal resolution parametric MRI is feasible for *in-vivo* monitoring and characterization of ischemia/reperfusion (I/R) induced AKI in rats. Fast T_2_*/T_2_ mapping was combined with a remotely controlled I/R model and a segmentation model based semi-automated quantitative analysis. This approach allowed tracking of T_2_*/T_2_ in the kidney during I/R with a high spatial and temporal resolution. Our study provides a technical advance in the field, as only snapshot images acquired before and after induction of injury were previously analyzed in I/R models [16]–[20].

The proposed segmentation model of the rat kidney served to reduce operator-induced variability compared to manually drawn regions-of-interest used in similar studies [16]–[19]. Taking into account that the histological borders among the kidney layers are rather undulating, extra care was taken to leave a generous spacing between the ROIs in the cortex and the outer medulla, and between the ROIs in the outer and the inner medulla to avoid partial segment effects. Thus, parametric MRI data of these ROIs can be attributed exactly to the respective morphological layer.

T_2_* and T_2_ changes were observed for all kidney regions shortly after induction of renal ischemia and during the initial reperfusion phase, which remained stable throughout the monitored reperfusion period. These findings could help to support the hypothesis that key events for the initiation of injury occur immediately after reperfusion, if a link to observations at later time points (e.g. 6–48 hours) can be established in future studies. In this study the surgical/experimental procedures were adapted for performing *in vivo* I/R experiments inside of an MR scanner; they do not allow for a recovery of the animal and additional MRI at later time points.

Considering the oxygenation-sensitivity of T_2_* (R_2_*) measurements during hypoxia, the immediate decline of T_2_* at the onset of ischemia, and the accordance with reports on invasively measured tissue pO_2_ during ischemia [12], it is reasonable to attribute the detected T_2_* (R_2_*) changes primarily to variations in intrarenal blood oxygenation. Still, T_2_* and T_2_ may only be surrogates for physiological parameters of interest, such as renal blood oxygenation. The influence of numerous other physical and physiological parameters on T_2_*/T_2_ variations *in vivo*, and in particular on the absolute values of these parameters, must be taken into account when comparing parametric MRI data with other quantitative measures of blood (or even tissue) oxygenation. For this reason it is conceptually appealing for future studies to combining invasive techniques and non-invasive MRI in a hybrid setup with the goal to calibrate, monitor and interpret parametric MR and physiological parameters by means of standardized interventions. Absolute values of baseline renal R_2_* were found to be lower in the inner medulla versus outer medulla and cortex, which is in agreement with previous reports [16], [24], [25]. This finding does not correspond with tissue pO_2_ levels measured by invasive methods [26], which showed the lowest pO_2_ levels in the inner medulla. One reason behind what seems to be a discrepancy is that the blood oxygenation dependence of R_2_* (T_2_*) does not relate to the deoxyhemoglobin/oxyhemoglobin ratio, but to the absolute amount of deoxyhemoglobin per tissue volume. Thus, besides being dependent on blood oxygenation, R_2_* (T_2_*) is influenced by the blood vessel volume fraction and the hematocrit. Both, vessel volume fraction and hematocrit are low in the inner medulla [27], [28].

In our study complete occlusion of the renal artery and vein were confirmed by MR angiography, rendering potentially confounding changes in global renal blood volume unlikely. Two possibly involved physiological factors are alterations in pH and fluid shifts. A significant drop in pH during renal artery occlusion in rats has been reported earlier [22]. By altering the hemoglobin oxygen saturation curve a pH reduction significantly influences the blood oxygenation effect on T_2_* [14]. To this end, simultaneous MRI mapping and conventional invasive measurements of oxygenation may allow further insights.

During hypoxia and ischemia MRI revealed significantly higher R_2_* indicating lower renal oxygenation. The observed increases in R_2_* during ischemia (180–500% of baseline) are comparable with previous reports [20] considering the difference in species (rat versus mouse model), magnetic field strength and analyses techniques. Parametric MRI monitoring, as established in our study, overcomes the limitations of snapshot data acquisitions at a few time points only and greatly enhances the assessment of spatio-temporal evolution of MR biomarkers – particularly in the initial reperfusion phase.

Our findings regarding R_2_* during I/R show similarities to invasively measured medullary pO_2_ time courses previously reported [12] and reflect impaired reoxygenation and hypoperfusion in the outer medulla after reperfusion which is related to the so-called “no reflow” phenomenon [29]. Parametric MRI indicated a strong persistent hypoxia in the outer medulla during reperfusion. The outer medulla is particularly susceptible to an ischemic insult due to high cellular metabolic rate (S3 segment of the proximal tubules, thick ascending limbs of Henle’s loop) is high and marked hypoperfusion and congestion persist during reperfusion, i.e. in the extension phase of AKI [30]. The R_2_* effects of hyperoxia on renal oxygenation were much smaller compared to hypoxia, which might be due to the already high arterial oxygen saturation of hemoglobin during normoxia. The amount of oxygen not bound to hemoglobin but dissolved in blood, which is expected to increase during hyperoxia, contributes only a rather small fraction to the overall oxygen supply.

Interleaved mapping of T_2_* and T_2_ may provide complementary information about the (patho)physiological conditions in the kidney as both MR parameters are affected by changes in blood oxygenation and water content to different degrees. Variations in the volume fraction of deoxygenated hemoglobin impact on the MR signal stemming from intra-vascular spaces of large vessels or capillaries as well as regions around these vessels. These intra- and extra-vascular signal components contribute differently to T_2_* and T_2_ [31], [32] and T_2_ is sensitive to water content [33]. Hence mapping of both parameters could allow for a more comprehensive assessment of renal oxygenation changes. The strong T_2_* and T_2_, changes observed in this study will inspire further explorations into MR methodology suitable for simultaneous T_2_*/T_2_ mapping.

In conclusion, continuous parametric MR monitoring of T_2_* and T_2_ throughout an I/R experiment in rats is feasible and provides a temporal resolution of approximately 80–100 seconds for each read-out resulting in 3 minutes for alternating T_2_*/T_2_ measurements. The proposed MRI approach enables the detailed assessment of *in vivo* changes in all kidney regions during ischemia and early reperfusion. Observations in the early reperfusion phase promise to offer new insights into the pathogenesis of ischemia induced AKI and might help to identify the timeline of key events responsible for development of hypoperfusion-induced renal damage. The established method of parametric MR monitoring holds the promise to be a useful investigational tool for other models of AKI such as x-ray contrast agent induced AKI, rhabdomyolysis-induced AKI, or sepsis-induced AKI.

## Supporting Information

Figure S1 55 T_2_* parameter maps of an entire ischemia reperfusion experiment. First row: After a baseline of 6 time points ischemia started at time point 7. Second row: onset of reperfusion after 45 minutes ischemia at time point 19. Rows 3–5: remaining time points of 100 minutes reperfusion. The parameter maps demonstrate immediate changes in T_2_* after onset of ischemia and onset of reperfusion.
(TIF)

Figure S2 **Corresponding T_2_ parameter maps to Figure S1.** First row: After a baseline of 6 time points ischemia started at time point 7. Second row: onset of reperfusion after 45 minutes ischemia at time point 19. Rows 3–5: remaining time points of 100 minutes reperfusion. Also in T_2_ immediate changes are visible directly after onset of ischemia and onset of reperfusion.
(TIF)

Table S1 **Summary of the hypoxia/hyperoxia and ischemia/reperfusion results.** Mean (±SEM) of T_2_* (R_2_*) and T_2_ (R_2_) of all regions-of-interest in the cortex (C1, C2, C3), in the outer medulla (O1, O2, O3) and inner medulla (I1, I2, I2). Shown are the values for the last time point in each experiment phase (e.g. end-baseline).
(TIF)
