# Supplementary figures and images for: High Temporal Resolution Parametric MRI Monitoring of the Initial Ischemia/Reperfusion Phase in Experimental Acute Kidney Injury
Source: PLoS One. 2013 Feb 28;8(2):e57411. doi: 10.1371/journal.pone.0057411 (PMC3585384; doi:10.1371/journal.pone.0057411)

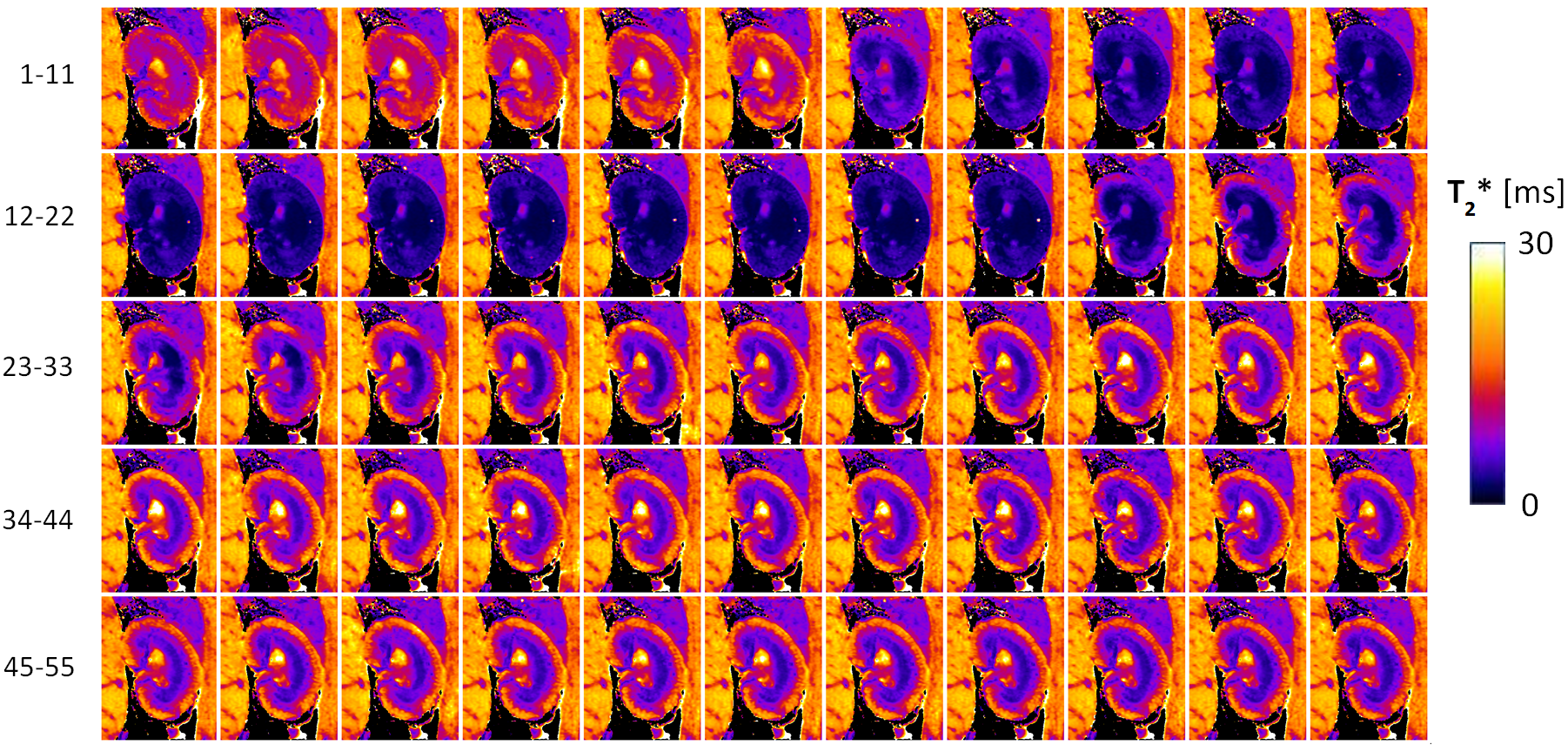

Supplement: Figure S1 — 55 T2* parameter maps of an entire ischemia reperfusion experiment. First row: After a baseline of 6 time points ischemia started at time point 7. Second row: onset of reperfusion after 45 minutes ischemia at time point 19. Rows 3–5: remaining time points of 100 minutes reperfusion. The parameter maps demonstrate immediate changes in T2* after onset of ischemia and onset of reperfusion. (TIF) [file pone.0057411.s001.tif]

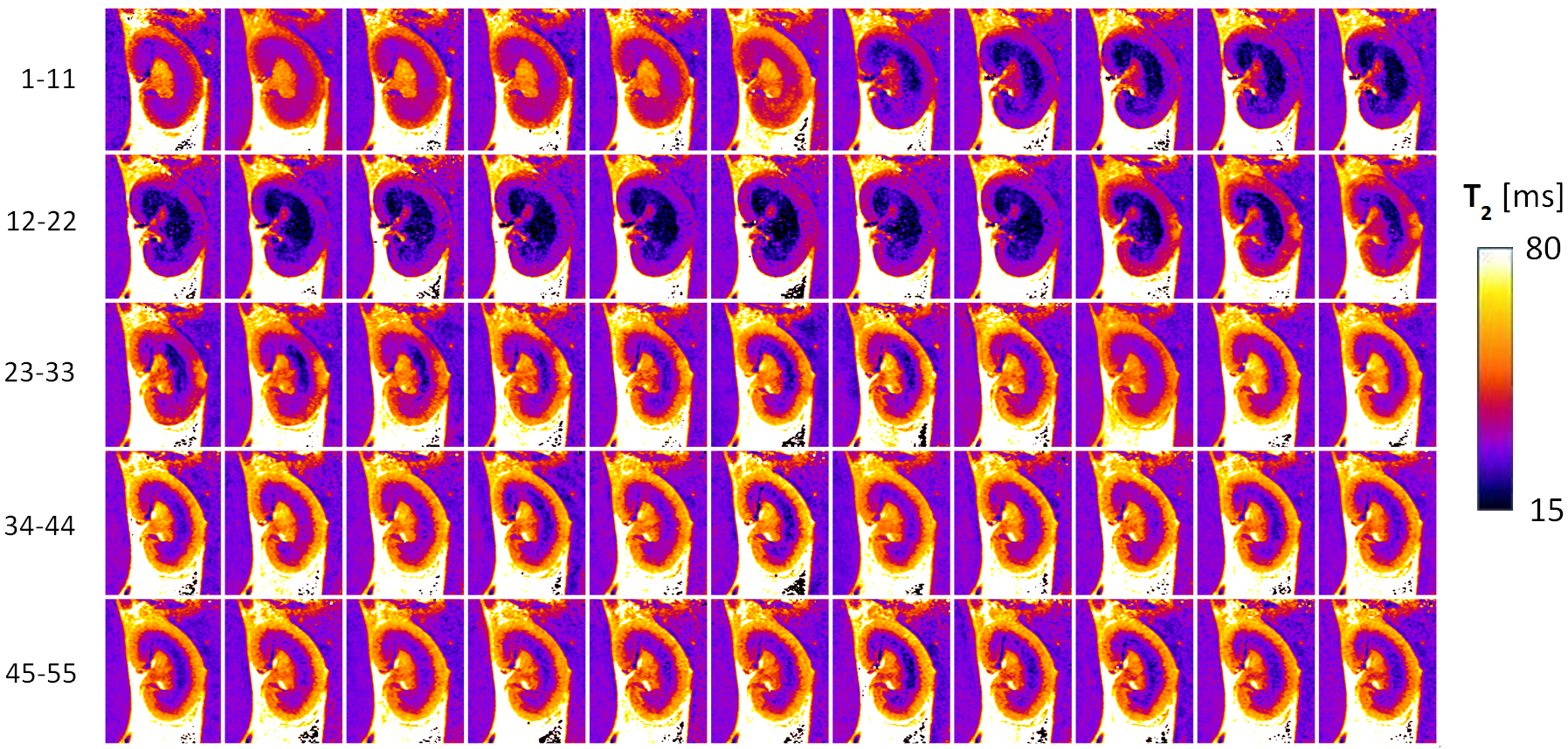

Supplement: Figure S2 — Corresponding T2 parameter maps to Figure S1. First row: After a baseline of 6 time points ischemia started at time point 7. Second row: onset of reperfusion after 45 minutes ischemia at time point 19. Rows 3–5: remaining time points of 100 minutes reperfusion. Also in T2 immediate changes are visible directly after onset of ischemia and onset of reperfusion. (TIF) [file pone.0057411.s002.tif]

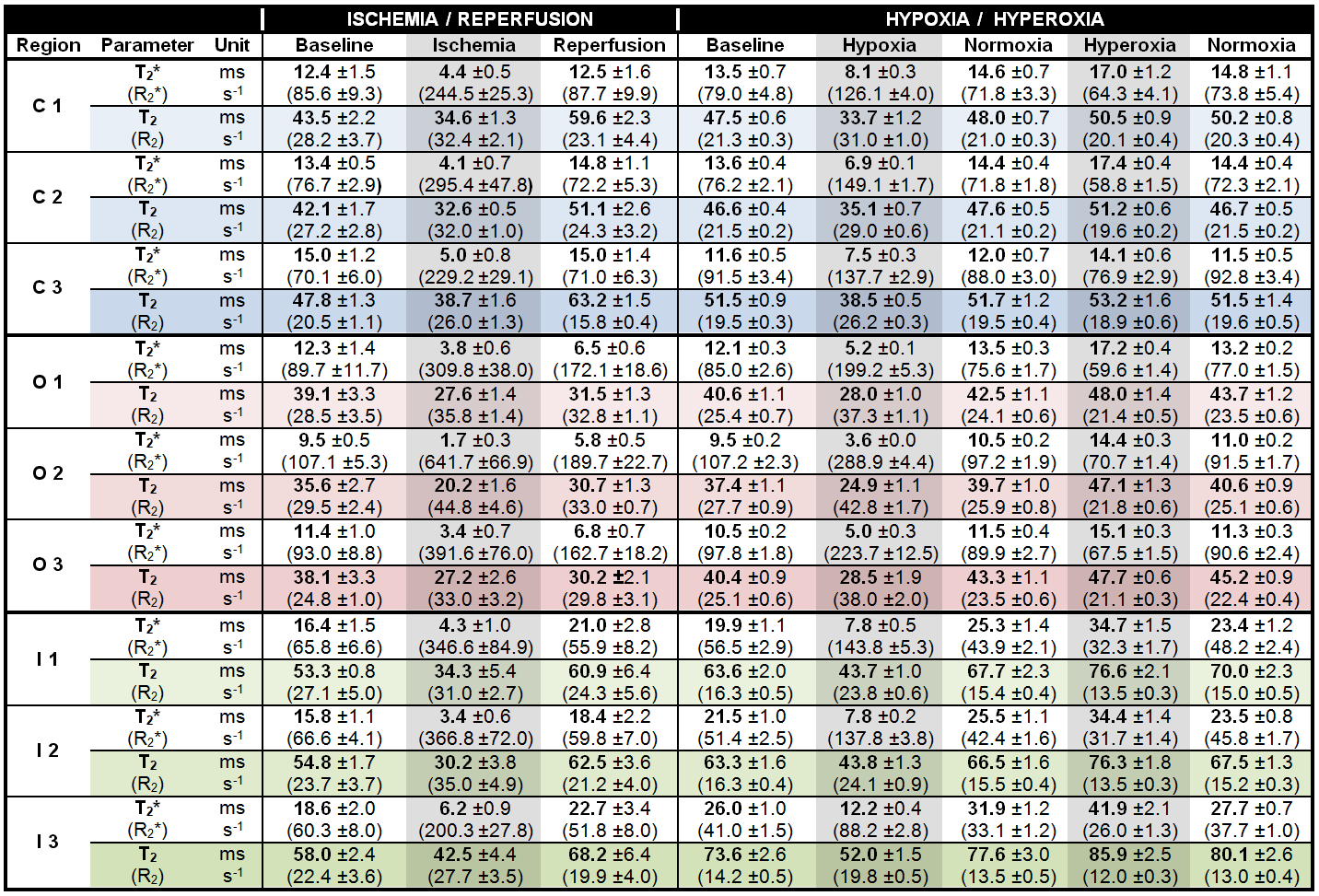

Supplement: Table S1 — Summary of the hypoxia/hyperoxia and ischemia/reperfusion results. Mean (±SEM) of T2* (R2*) and T2 (R2) of all regions-of-interest in the cortex (C1, C2, C3), in the outer medulla (O1, O2, O3) and inner medulla (I1, I2, I2). Shown are the values for the last time point in each experiment phase (e.g. end-baseline). (TIF) [file pone.0057411.s003.tif]
